# Adaptive Unsupervised Learning-Based 3D Spatiotemporal Filter for Event-Driven Cameras

**DOI:** 10.34133/research.0330

**Published:** 2024-04-01

**Authors:** Meriem Ben Miled, Wenwen Liu, Yuanchang Liu

**Affiliations:** ^1^ Department of Mechanical Engineering, University College London, London, UK.; ^2^ School of Automation, Nanjing University of Information, Science and Technology, Nanjing, China.

## Abstract

In the evolving landscape of robotics and visual navigation, event cameras have gained important traction, notably for their exceptional dynamic range, efficient power consumption, and low latency. Despite these advantages, conventional processing methods oversimplify the data into 2 dimensions, neglecting critical temporal information. To overcome this limitation, we propose a novel method that treats events as 3D time-discrete signals. Drawing inspiration from the intricate biological filtering systems inherent to the human visual apparatus, we have developed a 3D spatiotemporal filter based on unsupervised machine learning algorithm. This filter effectively reduces noise levels and performs data size reduction, with its parameters being dynamically adjusted based on population activity. This ensures adaptability and precision under various conditions, like changes in motion velocity and ambient lighting. In our novel validation approach, we first identify the noise type and determine its power spectral density in the event stream. We then apply a one-dimensional discrete fast Fourier transform to assess the filtered event data within the frequency domain, ensuring that the targeted noise frequencies are adequately reduced. Our research also delved into the impact of indoor lighting on event stream noise. Remarkably, our method led to a 37% decrease in the data point cloud, improving data quality in diverse outdoor settings.

## Introduction

Recent advancements in bioinspired and neuromorphic optical sensing have led to the introduction of event cameras, which offer a new way of capturing visual information, inspired by the visual perception mechanism as found in mammals. Although the benefits of taking advantage of and exploiting this technology are obvious, it has also brought new challenges, including the introduction of unique types of noise and the need for adapted processing and computer vision algorithms that can adequately process the asynchronous stream of data produced by event cameras.

**Fig. 1.**
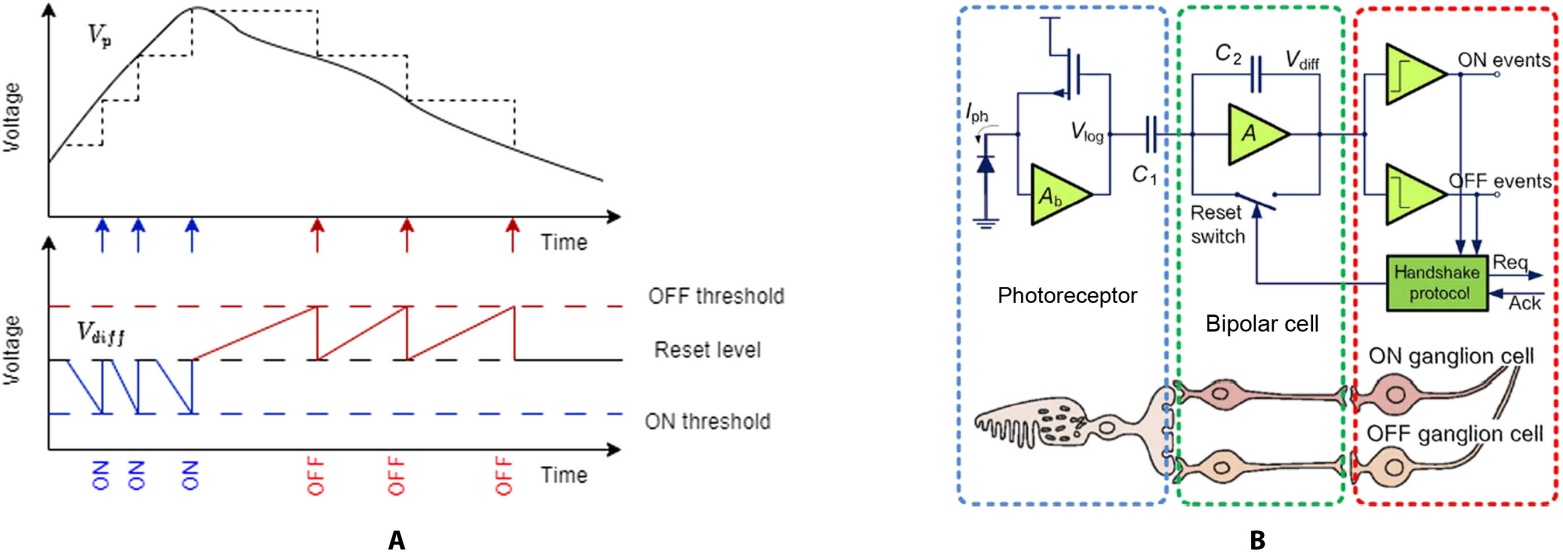
This is an example of a figure consisting of multiple panels. (A) Pixel events are triggered when the logarithmic intensity *V*_p_ variation of illuminance surpasses a certain threshold. If the log intensity *V*_diff_ exceeds the threshold, the event polarity is positive (ON); the event polarity is negative (OFF) when the log intensity falls below the threshold. (B) The 3-layer human retina model, along with its corresponding DVS pixel circuitry, represents a bioinspired approach to vision processing. This model mimics the structure and function of the human retina, which consists of 3 primary layers: the photoreceptor layer, bipolar cell layer, and ganglion cell layer [37].

An event camera, also known as a bioinspired visual sensor or silicon retina, operates by detecting changes in pixel intensity (brightness) over time, as opposed to capturing images at a fixed frame rate as with traditional cameras. As illustrated in Fig. 1A, a pixel generates and transmits a signal (event) when the variation of logarithmic intensity of illuminance exceeds a specified threshold. This innovative approach to visual sensing endows event cameras with several advantages, including high temporal resolution (microseconds), high dynamic range (above 120 dB), low power consumption (in the order of milliwatts), and low latency (in the order of microseconds) [1]. This makes them well-suited for specific applications under conditions where normal, standard hardware would be required to operate and function beyond its design limitations in fields such as robotic navigation [2], object tracking [3], surveillance systems [4], human–computer interaction [5], and dynamic obstacle detection [6].

An event camera operates by asynchronously generating events for each pixel whenever a change in illuminance intensity exceeds a predefined threshold. Each event contains information with regard to the pixel’s location, the sign of the intensity change (polarity), and the precise time stamp in millisecond at which the change occurred. This approach results in a sparse, time-continuous stream of events, providing a rich representation of the dynamic visual scene. The pixel structure mimics the functioning of the human retina (Fig. 1B) as the device is based on the functioning of the magnocellular pathway [7]:

• Photoreceptor: The photoreceptor is responsible for detecting and converting incoming light into an electrical signal proportional to the light intensity.
• Comparator/ON–OFF bipolar cell: The comparator compares the photoreceptor’s output to a threshold value. When the difference between the current and previous photoreceptor output exceeds the threshold, an event is generated. This threshold can be either fixed or adaptive, depending on the camera’s design.
• Digital circuitry/retinal ganglion cells (RGCs): The digital circuitry processes the event data, typically encoding the pixel location (*x*, *y*), the polarity of the intensity change (increase or decrease), and a time stamp that identifies when the event occurred. This information is then transmitted asynchronously to the processing unit.

As seen in Fig. 1B, the initial stage converts first the photocurrent into a voltage that is proportional to the logarithm of the light intensity:

$$V_{\text{log}}=V_{\text{DC}}+A_{v}U_{T}\ln\left(I_{\text{ph}}\right)$$ (1)

where *U_T_* is thermal voltage, *A_v_* is a voltage gain factor, and *V_DC_* is a light-independent direct current. Then, in the second stage, the logarithmic voltage (*V*_log_) is amplified by the factor *C*_1_/*C*_2_, with the charge accumulated at *C*_2_ being reset whenever the difference in voltage (*V*_diff_) varies more than a fixed threshold established by the comparators. As a result, each pixel generates a signed asynchronous output whenever the change between time *t*_2_ and *t*_1_ in light intensity, represented by ln(*I*_ph_(*t*_2_)) −  ln (*I*_ph_(*t*_1_)), exceeds a threshold.

It is important to note that during real-world field experiments, event cameras tend to produce outputs with significant levels of noise. This noise can markedly impair the performance of the detection and recognition algorithms being used, as pointed out by prior research [8]. Under such conditions, the absence of reliable noise models or classifications for event camera data makes it challenging to appropriately assess noise levels. As a consequence, tackling the issue of noise processing in event cameras can manifest itself as a critical problem that warrants further investigation.

Event noise can be divided into background noise (BA) [9] and temporal noise [10]. Background activity events are triggered without any brightness or movement changes. The main causes of BA noise are thermal noise and current leakage of switches. Unlike BA, temporal noise is not related to hardware limitations but to the quantum nature of light, which is caused by the inherent randomness in photon arrivals at the sensor, leading to variations in the detected signal over time [11].

Most studies examining filtering events from noise have relied on spatiotemporal correlation for event emission, focusing predominantly on the local vicinity or small area surrounding the event for filtering, without considering the broader picture. For example, in [12], the authors focused on addressing background activity noise by proposing a spatial filter that uses local neighborhood information around each event to identify and suppress noise. The temporal filter considers time intervals between consecutive events at each pixel, detecting and removing events with irregular temporal patterns to further reduce BA. Both [9,13] constructed a probabilistic undirected graph model to distinguish between effective events (exhibiting temporal and spatial regularity) and noise events (showing randomness). The denoising issue is then transformed into a probability maximization or minimization problem. In [14], a new denoising method for dynamic vision sensors (DVSs) was introduced, leveraging augmented spatiotemporal correlation. The researchers proposed 2 filters: the overlapped event filter, which detects and removes high-frequency noise using an event density map, and the transient event filter, which eliminates low-frequency noise through spatiotemporal neighborhood consideration. In [15], the authors generated an event address map using spatiotemporal correlation filtering with both small and large neighborhoods to eliminate noise. They then applied median filtering, followed by opening operations to further filter background activity and restore the missing events (false negative).

In recent studies, researchers have explored a supervised machine learning approach to addressing the problem of denoising-event-based data [16,17]. The authors in [18] introduced an event probability mask (EPM) that serves as a proxy for ground truth labels, which is computed from active pixel sensor (APS) and inertial measurement unit (IMU) measurements. They developed a event denoising convolutional neural network called EDnCNN, designed to perform a hypothesis test to determine whether a specific event corresponds to an actual log-intensity change or whether it is merely noise. The training data for EDnCNN consist of DVS events along with their respective EPM labels. Once the EDnCNN is trained, it can classify events without relying on APS, IMU, stationary scenes, or constrained camera motion.

Evaluating the performance of event camera denoising algorithms is a challenging task due to the absence of a unified benchmarking method and annotated ground truth. To address this issue, several studies have attempted to standardize evaluation metrics. In [19], the authors introduced the event denoising dataset multilevel benchmark (E-MLB) dataset, consisting of 100 scenes with a variety of motions, corresponding APS frames and IMU data, including challenging sequences such as low light and special weather conditions. They also proposed a new metric called event structure ratio, which combines the normalized total sum of squares and the penalty coefficient to evaluate the denoising performance. Normalized total sum of squares measures the relative contrast of the scene, and penalty coefficient penalizes excessive denoising. The efficacy of the event structure ratio metric is underscored by its ability to consistently measure denoising performance regardless of the number of events in a batch. Specifically, even as the number of events (indicative of scene activity) and associated noise increase, the event structure ratio metric maintains accuracy, becoming more discerning in higher noise environments. The authors demonstrated the effectiveness of the event structure ratio metric on both synthetic and real data. In [14], the authors proposed the event denoising accuracy metric based on motion compensation of events and the calculation of the DVS response probability map. The event denoising accuracy metric was obtained by comparing the logarithmic of the plausibility of the event stream before and after denoising and was used to evaluate the performance of denoising algorithms. This evaluation method requires the camera to move relative to a static scene and information about the DVS trajectory. In conclusion, while the mentioned denoising evaluation methods show potential, they have yet to be conclusively proven in practical scenarios. Further testing and validation are needed.

In this study, we propose treating event data as a 3-dimensional (3D) discrete signal, moving away from the conventional approach where events are transformed into standard images through frame generation or event accumulation. In traditional methods, events are accumulated over fixed periods to generate frames akin to those of traditional cameras. This results in time-integrated images that represent collective scene changes. While this transformation enables the use of existing 2D image processing techniques for denoising, it can compromise the unique temporal resolution and high dynamic range inherent in event data. Our 3D methodology is designed to retain and emphasize these granular temporal intricacies rather than condensing them into static frames. Thus, in this research, we pioneer a comprehensive approach to event data processing by delving deeply into the origins of noise, from photon fluctuations to the intrinsic electronic noise of a single pixel detected by the event camera. Given the inherent challenge in discerning noise from ground truth in event data, our innovative approach reframes denoising as a data compression task. Specifically, we target and remove frequencies predominantly associated with noise, based on our extensive analysis.

To tackle these identified noise frequencies, we have implemented a novel bioinspired 3D filter grounded in unsupervised learning. Indeed, the human retina filters visual noise through specialized cells and neural circuitry, enhancing signal clarity and reducing irrelevant information before processing in the brain. In term of signal processing, the retina’s mechanisms can be likened to 3D clustering and data compression in digital systems. Just as temporal and spatial summation in the retina merge signals over time and space to filter noise, 3D clustering groups similar data points in a 3D space, simplifying complex datasets. Meanwhile, just as the retina condenses visual data for efficient transmission to the brain, data compression reduces file sizes for faster storage and transfer, preserving the most critical information. Both systems prioritize clarity and efficiency in their respective domains. Further distinguishing our methodology, the filter parameters undergo dynamic adjustments through the neuroscience-inspired population activity (PA) method. This ensures that our filter remains adaptive, continuously optimizing performance in response to the shifting data landscape and dynamic environmental variables captured by the camera.

A pivotal aspect of our evaluation strategy involves the introduction of a 1D discrete fast Fourier transform (FFT) for events. By using this FFT, we can meticulously track shifts in noise frequencies, offering insights into the effectiveness of our compression-based denoising method. This, in tandem with visual assessments conducted on outdoor event data across various lighting conditions, provides a comprehensive evaluation of our approach’s efficacy in enhancing event data quality. Moreover, our research provides an exhaustive study on the influence of various outdoor lighting scenarios on noise, further enhancing our understanding of noise origin and its mitigation in dynamic environments.

## Results

### Impact of ambient lighting on event data noise

#### Performance under intense natural luminance

Our analysis of event cameras under various outdoor lighting showed distinct performance patterns, most notably in bright sunlight. This gave us deeper insight into the technical issues faced under these conditions.

**Fig. 2.**
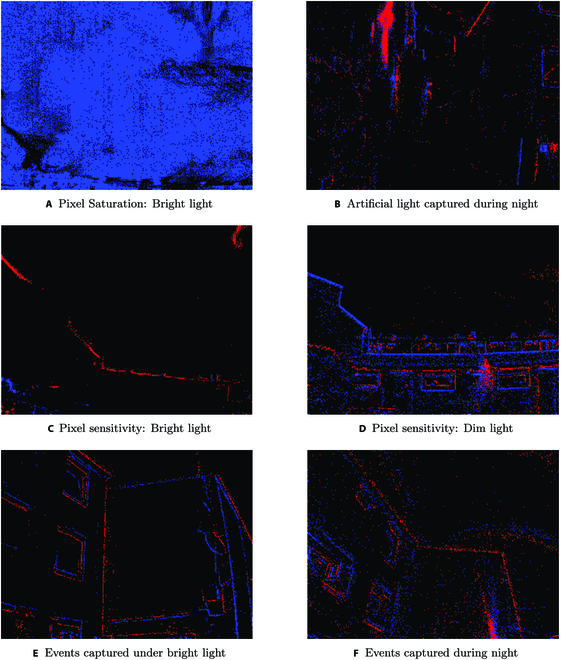
Impact of natural and artificial lighting on unfiltered raw event data. (A) Pixel saturation: bright light; (B) artificial light captured during night; pixel sensitivity: (C) bright light and (D) dim light; and events captured (E) under bright light and (F) during night.

The first impact is pixel saturation: The sun’s potent luminance had resulted in widespread pixel saturation across the event camera sensor. The graphical representation clearly demarcates regions where the pixels are overwhelmed by the influx of photons, quickly exceeding their electron storage limits and as a result generating a false positive. In fact, once this threshold is crossed, additional photons do not amplify the accumulated charge in these pixels. This persistent state essentially means that the pixel remains unresponsive, or blind, to subsequent brightness variations until the overall luminance recedes to less than the saturation limit. In typical scenarios, these saturated pixels would simply lead to overexposed regions in an image. However, the ramifications for event cameras are more nuanced. Referring back to Fig. 2A where areas directly exposed to the sun manifest a significant increase in event generation (all the pixels were triggered at the same time), the sudden burst of events breaks the steady stream of data, creating gaps in the recorded information. This unevenness can lead to a distorted or incomplete view of moving scenes, which could be problematic for applications that rely on constant and real-time event data.

The second effect caused by bright sunlight is the diminished pixel sensitivity. Under the influence of intense brightness, the operational efficacy of the pixels in event cameras becomes markedly compromised. In less extreme lighting scenarios, these cameras excel in discerning even the most subtle shifts in luminance, ensuring that every meaningful change is registered as an event. However, under overly bright conditions, the intrinsic sensitivity of the pixels deteriorates. The challenge that arises is rooted in the pixel’s diminished capacity to distinguish between varying brightness levels when overwhelmed with light. As the ambient background brightness increases, the subtle variations in light intensity that would otherwise trigger events become nearly indistinguishable from the dominant light source. As a result, the camera may register and return false negatives as seen in Fig. 2C, resulting in situations where significant brightness variations occur but are overshadowed by the prevailing luminance, causing the camera to not detect and register them. These false negatives can be particularly problematic. Since events might not be generated when ideally they should be, crucial details of the environment, as captured under dim light conditions in Fig. 2D, can remain uncaptured under intense natural luminance. This means that in the presence of a strong light source, such as direct sunlight, the camera could fail to capture important nuances of the surroundings, creating gaps in the data and potentially compromising the overall fidelity and accuracy of the camera’s output.

#### Performance in dim and dark outdoor settings

Our analyses extend to the event camera’s functioning in dim and dark outdoor environments, specifically during evenings, after sunset, and night devoid of bright sunlight. The presence of artificial lighting during these periods presents unique challenges, affecting not only the event data but also postprocessing techniques such as denoising algorithms.

Artificial light sources, in dark outdoor scenarios, result in a pronounced spatial disparity in the distribution of event data. In other words, artificial light affects data clustering performance. This disparity is most evident as regions of high data density near the light source. Such variance is influenced by the nonuniform photon emission patterns of artificial lighting as their brightness varies across their emission spectrum and, combined with the asynchronous characteristic of event cameras’ response of event cameras, often leads to significant and instantaneous changes in intensity. Such rapid changes, especially if a light source has a flickering behavior, can lead to a cascade of events within a relatively brief time frame. As seen in Fig. 2B, the artificial light (top left of the picture) has higher event density compared to the surroundings. The challenge arises when the algorithm HDBSCAN (hierarchical density-based spatial clustering of applications with noise), which is tailored for identifying and segregating data clusters, encounter this data landscape. The inherent logic of HDBSCAN is based on determining core samples of high density and extrapolating clusters from these high-density sample. In situations where there is an extreme contrast of data densities, dense event regions near artificial lights versus sparse ones elsewhere, the algorithm can falter. This high gradient between regions might cause the significantly less denser area to be perceived as an outlier or even be categorized as noise, especially if the dense regions lack substantial connectivity to the broader data structure. The feature space, which shows the event data’s characteristics, gets noticeably altered near artificial lights. Elements such as event frequency, temporal consistency, and spatial coherence deviate drastically between dense and less dense regions. The pronounced difference produced by this contrast in light density further compounds the difficulty for HDBSCAN in making accurate classifications. Moreover, HDBSCAN’s reliance on local density metrics for cluster formation is a source of weakness when applied to scenarios with such diverse lighting conditions. The sharp variations in event densities around artificial light sources can adversely affect the algorithm’s assessment of the local region of influence, potentially leading to the derivation of inaccurate cluster boundaries.

As illustrated in Fig. 2E and F, which align with our preliminary calculations, environments with dim and dark lighting conditions inherently elevate the noise level within the captured event data. This augmentation in noise becomes even more apparent when visually assessing sequences recorded under varying light conditions ranging from bright to dim and dark. Considering our data acquisition process was closely controlled, whereby all sequences were recorded using the same camera within a consistent environment and at a steady velocity, it is therefore justifiably feasible to draw comparative insights. Despite the absence of a clear ground truth for direct validation, our comparative method offers a reliable way to identify and measure the noise increase in less favorable lighting conditions.

**Fig. 3.**
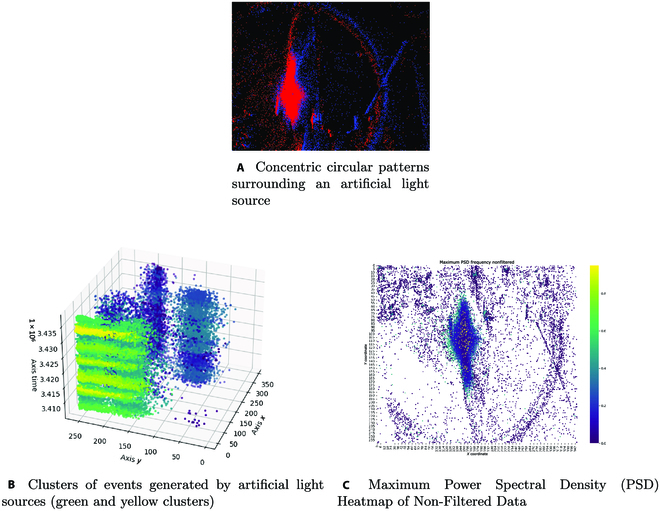
Impact of artificial lighting on event generation. (A) Concentric circular patterns surrounding an artificial light source. (B) Clusters of events generated by artificial light sources (green and yellow clusters). (C) Maximum PSD heatmap on nonfiltered data.

Moreover, the event data distinctly showcases concentric circular patterns encircling the light sources as seen in Fig. 3A. These concentric patterns, while often being imperceptible to the human eye, are genuine manifestations of light intensity variations. Their origin can be traced back to effect of diffraction when light interacts with the camera’s lens system or from the spatial intensity gradients radiating from the light source. Such nuances are picked up by the event camera due to its acute sensitivity to change in the light intensity. However, the concentric circular patterns’ presence creates challenges in data processing. Event-based algorithms, especially those tailored for tracking and recognition, rely heavily on distinguishing authentic features data from BA. The concentric circular patterns, being real intensity variations, can adversely impact these algorithms, potentially introducing inaccuracies or false positives. In essence, while these circular patterns are true indicators of brightness changes, they can inadvertently detract from the optimal performance of particular event-based algorithms.

Finally, artificial light sources, when interrogated through the lens of event cameras, present unique event distribution patterns in the temporal–spatial domain. The events generated by these light sources exhibit well-defined clustering behavior in 3D space. Such patterns arise because of the periodic nature of artificial light emissions, often caused by the AC powering. This periodicity leads to light intensity modulations at a frequency typically related to the AC frequency (e.g., 50 or 60 Hz in most regions). These periodic fluctuations culminate in the formation of compact, equidistant event clusters as seen in Fig. 3B, as the camera captures the rhythmic intensity variations of the light source. When we delve into the maximum power spectral density (PSD) map, these clusters translate into pronounced peaks as seen in Fig. 3C where the maximum frequencies are located at the center of the artificial light source (shown as yellow dots). This manifestation is consistent with the Fourier transform principles, where periodic signals lead to sharp spikes when presented in the frequency domain. The resultant peaks from artificial light sources, in this spectrum, appear more frequently than those corresponding to the broader environment. These higher frequencies are indicative of rapid changes in light intensity, typical of many artificial sources. Photodiodes in the event camera, sensitive to minute changes in photon influx, trigger events more frequently when faced with such rapid oscillations. While these sharp and periodic frequency components provide valuable insight into the nature of the light source, these components pose challenges. Any applied algorithms need to discern these high-frequency components, associated with artificial light, from other potential high-frequency noise or events in the scene, to help ensure accurate data interpretation without importing classification errors.

**Fig. 4.**
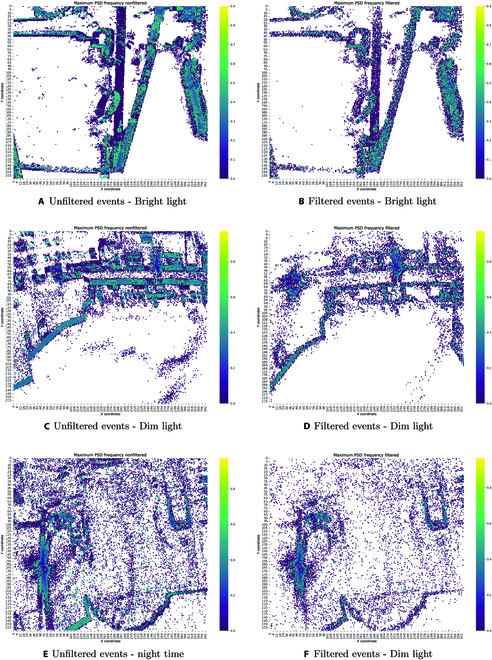
Denoising algorithm performance under varying illumination conditions with fast moving camera’s frame: maximum PSD heatmaps generated from events within a 10-ms time frame. Unfiltered events: (A) bright light, (C) dim light, and (E) nighttime. Filtered events: (B) bright light, (D) dim light, and (F) nighttime.

To assess the effectiveness of our denoising and data compression algorithm, we will undertake a detailed analysis of the frequency patterns in the data. This involves a comparison between the frequencies evident in the initial, unfiltered dataset and those present after filtering. By collecting data under a spectrum of lighting conditions, we aim to ascertain the algorithm’s capability to adapt and maintain consistency across a range of external illumination scenarios. Thus, to analyze the frequency characteristics of the event data, a maximum PSD frequency map was generated. The PSD is a fundamental tool in signal processing that quantifies how the power of a signal is distributed over its frequency components and typically expressed in units of power per frequency interval. In other terms, it offers a profile of the signal’s strength across different frequencies. For our event data, we generated a maximum PSD frequency map. This map effectively pinpoints where the majority of the signal’s energy is concentrated in terms of frequency. By comparing the PSD of the raw event data to that of the filtered data, we can gauge the effectiveness of our denoising algorithm, specifically in its ability to attenuate or remove frequencies associated with noise, while preserving those pertinent to the actual event signal. The comparative analysis thus aids in ensuring that our filtering approach maintains data integrity, especially under varying light conditions. This map is constructed by first applying the FFT to each individual pixel’s temporal event stream. The FFT is a widely used technique that transforms time-domain signals into frequency-domain representations, which allows for the isolation and examination of different frequency components of the data. After applying the FFT, PSD is computed for each pixel. For our study, we used the PSD to break down the signal’s strength across its frequencies. We calculated the PSD by squaring the FFT results and then dividing by the signal’s length. This method helps standardize the data, making it easier to compare between different pixels and scenes, even if they have varying amounts of information. Here, the term power is essentially the activity level at each pixel, indicated by the rate of events. In the maximum PSD frequency map, we pinpoint the highest frequency value for each pixel. Essentially, this shows us that frequency is most dominant for every individual pixel. By doing this for all pixels, we get a clear picture of the main frequency patterns across the whole scene. In our frequency analysis, we discerned a pronounced concentration of noise within the low-frequency domain, as delineated by the preponderance of purple markers in Fig. 4A, C, and E. The omnipresence of these purple indicators, particularly in sparse regions of the frequency map, corroborates the hypothesis that noise is predominantly confined to these lower frequencies. While a quantitative substantiation remains elusive because of the absence of an established ground truth, the visual manifestations serve as a potent heuristic. It is imperative to elucidate that not every manifestation in the low-frequency domain can be ubiquitously classified as noise. Nonetheless, our prior knowledge of the actual environment facilitates heuristic demarcation of probable noise-centric areas. This observed trend matches what we often see in many real-world systems, where random changes or slow shifts in the environment typically produce noise in the low-frequency parts of the signal. Interestingly, our data show that the important environmental outlines and features, crucial for efficient data compression, mainly appear in the mid to high-frequency range, as shown by the green–blue markers. Indeed, upon inspecting the maximum PSD frequency map of the filtered data, a notable outcome is that the denoising/data compression algorithm effectively attenuates these low-frequency components (shown as purple dots) while keeping the important features (green–blue dots) as seen in Fig. 4B, 4D, and 4F. In essence, the denoising process acts as a high-pass filter, allowing high-frequency components, which correspond to rapid intensity changes and genuine events, to pass through, while substantially reducing the power of low-frequency noise. This high-pass filtering property of the denoising and data compress algorithm is a critical feature. It ensures that the essential details captured by the camera, which are characterized by high-frequency components due to their transient nature, are preserved in the denoised data. Meanwhile, undesirable low-frequency noise, which does not contribute to the meaningful interpretation of the scene, is significantly minimized, enhancing the clarity and reliability of the event data for subsequent processing and analysis.

As mentioned above, it is important to note that not all components in the low-frequency range equate to noise; some genuinely represent gradual changes in scene intensity. These might be due to shifting shadows or clouds altering the lighting, and they might sometimes be mistakenly addressed as noise by the denoising algorithm. This nuance partly justifies viewing our denoising technique as a compression method. This approach selectively retains essential scene structures by reducing low frequencies, as illustrated in Fig. S4. The 3D clustering method we have used effectively preserves significant data clusters while filtering out BA, as evident in Fig. S4A and B. This effectiveness is further showcased in the accumulated images, where the filtered image Fig. S4D predominantly emphasizes edges and primary features, making it more defined than the original (Fig. S4C). By focusing on higher-frequency components, our algorithm ensures retention of pivotal scene changes vital for applications like motion analysis or object tracking.

By filtering out slower, possibly redundant variations, we not only refine the data’s clarity but also reduce its volume, optimizing it for subsequent processing stages. In evaluating the performance of our data compression algorithm under various lighting conditions, we observed the following results:

• Bright light: From an initial 40,000 events per iteration, approximately 14,783 were identified as noise, translating to a denoising efficiency of 36.95%.
• Dim light: With the same 40,000 events per iteration, around 15,032 were classified as noise, achieving a 37.58% denoising efficiency.
• Night (utilizing artificial light): Of the 40,000 events, about 14,893 were pinpointed as noise, yielding a 37.23% denoising efficiency.

As indicated above, we managed to cut down the dataset’s size by approximately 37% across various lighting scenarios without sacrificing essential scene details. The core of our method is thus about preserving the dataset’s key structural elements and crucial features, all while delivering a more concise representation that accentuates rapid scene changes over minor, slow ones.

### Impact of velocity and lighting on PA dynamics

In our experiments, we methodically varied the velocity of the camera to study its influence on the event PA across different lighting conditions: bright, dim, and dark. We observed a notable correlation between camera velocity and event PA. As the camera’s velocity increased, we registered a corresponding increase in the event PA. This trend was consistently observed, irrespective of the ambient lighting conditions and various velocities under which the camera was operating as seen in Fig. S3. During slow camera movements, the activity levels remained consistent across different lighting conditions, as illustrated in Fig. S3B, C, and F. One primary factor behind this uniformity is the introduction of artificial lighting in dark scenarios, a topic elaborated upon in the “Performance in dim and dark outdoor settings” section. Such supplementary illumination becomes imperative for event cameras operating in low-light environments. Without it, the camera’s efficiency dips significantly, potentially necessitating the deployment of alternative sensors like infrared or light detection and ranging. The strategic use of artificial light effectively bridges the disparity in event activity across different ambient conditions, ensuring a stable event generation rate during slow camera movements.

Furthermore, regardless of the lighting condition, there was a discernible correlation between an increase in camera speed and a rise in PA as illustrated in Fig. S3A, C, and E. This trend is not just an incidental observation. This relationship is particularly significant when considering clustering algorithms like HDBSCAN. The PA effectively captures the dynamism and density of the events in the captured data. By utilizing this metric, it becomes feasible to dynamically adjust the parameters of the HDBSCAN clustering algorithm. Specifically, a heightened PA, which signifies denser or more frequent events, can inform the algorithm to adapt its clustering granularity or sensitivity. In essence, this ensures that the clustering remains responsive and tailored to the nuances of the data, optimizing the segregation of significant events from potential noise or less relevant data points.

In contrast, during faster camera movements, we observed divergent patterns. Nighttime conditions, as seen in Fig. S3B, exhibited a more pronounced activity compared to bright conditions showcased in Fig. S3F. This can be attributed to 2 factors: First, the swift motion seems to diminish the noise-amplifying effects of artificial lighting. Second, under bright conditions with increased velocities, the challenge of event generation is compounded by saturation, as mentioned in the “Performance under intense natural luminance” section. The frenetic pace does not grant the pixels enough recuperation time, thus affecting the efficacy of event detection.

### Qualitative and quantitative evaluation of our algorithm relative to established state-of-the-art denoising techniques

To evaluate the efficacy of our 3D adaptive denoising algorithm, we utilized the E-MLB dataset [19]. This dataset plays a crucial role in examining the correlation between noise levels and light intensity as neutral density (ND) filter was placed over the event camera to emulate varying lighting conditions. Each scene was initially captured under natural lighting conditions. Subsequently, the process was repeated with the addition of an ND filter in front of the DVS. Three types of ND filters were used with distinct transmittance levels (1/4 and 1/16), labeled as ND4 and ND16, respectively. To further enhance the diversity of lighting conditions, the E-MLB dataset includes captures extending from day to night, ensuring a broad spectrum of natural lighting scenarios.

For the comparative analysis of our algorithm, we selected 6 of the most representative event denoising methods. It is important to note that we deliberately excluded denoising algorithms based on supervised machine learning from this comparison. The rationale behind this exclusion lies in the high computational demand of such methods and the necessity of labeling extensive datasets under multiple lighting conditions. Among the chosen comparative methods, KNoise [20] and DWF [12] adhere to similar denoising principles, using background activity filter. Background activity filter operates by counting the density of each incoming event within an 8-pixel neighborhood over a specified time interval. It then applies a predetermined threshold to filter out noise events. time surface (TS) [21] methodology transform sparse event streams into dense representations. TS achieves this by converting the Dirac time function into a logarithmic decay representation, forming a structured manifold known as the time surface. This process involves removing events that disrupt the time surface’s smoothness. EvFlow [22] utilizes local plane fitting for gradient calculation and optical flow determination, filtering out events with abnormal flow for denoising. YNoise [23], on the other hand, denoises by passing events with high spatiotemporal density, assessing each event’s density in its context. Finally, reclusive event denoisor (RED) [19] denoises using state space models for temporal smoothing and a fast Deriche blur algorithm for spatial processing. It filters events based on their density in a spatiotemporal context, retaining those with density above a certain threshold.

#### Quantitative evaluation

In our study, we conducted a comprehensive quantitative evaluation of various denoising methods, including our own, as seen in Table. This analysis was crucial to assess the computational efficiency and effectiveness of our approach compared to the state of the art. We compared the peak memory use (PMU), computational time (CT), and percentage of data reduction (DR) across different denoising algorithms. Our algorithm demonstrated significant efficiency in data reduction, achieving the second-best performance with a 40% reduction rate, as underlined in TableA. It is important to note that the best and second-best performances in terms of data reduction were determined while also considering the tendency of other algorithms to overdenoise. Our algorithm strikes a balance between effective noise reduction and preserving essential data, avoiding the common pitfall of overdenoising and losing substantial information that was observed in other algorithms with higher data reduction percentages. In addition, EvFlow, another focal point of our analysis, while showing a comparable PMU to our method and others, had a significantly higher CT, being 145 times greater than that of our algorithm. This marked difference highlights the superior processing efficiency of our approach. In terms of PMU, all methods exhibited similar performance, indicating a general efficiency in memory usage across the board. However, the stark contrast in CT, particularly for EvFlow, underscores the need for balanced optimization between memory use and processing speed in denoising algorithms.

**Table.** Quantitative evaluation of our algorithm relative established denoising methods

(A) Comparative metrics of computational resources: PMU, CT and % DR. We mark the best in boldface and second best in underscore.			
	**PMU (MiB)**	**CT (s)**	**% DR**
Ours	192.43	72.06	40
YNoise	171.57	0.098	58
KNoise	188.36	0.159	76
EvFlow	186.06	10,442.706	**11**
RED	181.24	33.412	75
DWF	167.31	0.316	52
TS	178.44	0.436	84
(B) The mean event structure ratio for the 2 best denoising methods (our algorithm and EvFlow) on E-MLB dataset. We mark the best in boldface.	
		**EvFLOW**	**Ours**
Daytime	ND00	3.2549	**3.2702**
	ND04	**3.1902**	3.1882
	ND016	2.9230	**3.0125**
Night	ND00	2.4989	**2.6730**
	ND04	1.5284	**1.9203**
	ND16	1.4342	**1.9134**

To finalize our comprehensive quantitative assessment, as presented in TableB, we meticulously compared the denoising performance of our 3D adaptive algorithm with that of EvFlow. This comparison was pivotal, given that both algorithms were specifically selected for their proficiency in effective denoising while avoiding the common issue of overdenoising. Indeed, the event structure ratio [19] is a sophisticated, nonreference metric for event denoising. It quantitatively measures the structural intensity of events, providing a robust assessment of denoising quality. This metric is particularly valuable as it is independent of the number of events and their projection direction, offering an unbiased comparison. In the context of denoising, a higher event structure ratio value is indicative of superior performance.

Our analysis, based on the event structure ratio values across different lighting conditions simulated with ND filters, revealed key insights. Both algorithms showed a decrease in denoising performance from daylight to darker conditions, correlating with increased noise levels in low-light environments. However, this degradation in performance was more pronounced for EvFlow compared to our algorithm, indicating our method’s enhanced resilience to noise. Furthermore, the event structure ratio values during daytime conditions, under ND filters such as ND00, ND04, and ND16, illustrated that our algorithm either matched or exceeded the performance of EvFlow. In more challenging nighttime scenarios, our algorithm consistently registered higher event structure ratio values across all ND filter settings. This consistent superiority in event structure ratio values, particularly under low-light and noise-intensive conditions, highlights the robustness of our algorithm.

This detailed quantitative comparison underscores the effectiveness of our 3D adaptive denoising algorithm in maintaining the structural integrity of events, even in diverse and challenging lighting conditions. It confirms our algorithm’s advanced capability in handling complex denoising tasks while preserving essential event details, thus establishing its superior performance in varied denoising scenarios.

#### Qualitative evaluation

**Fig. 5.**
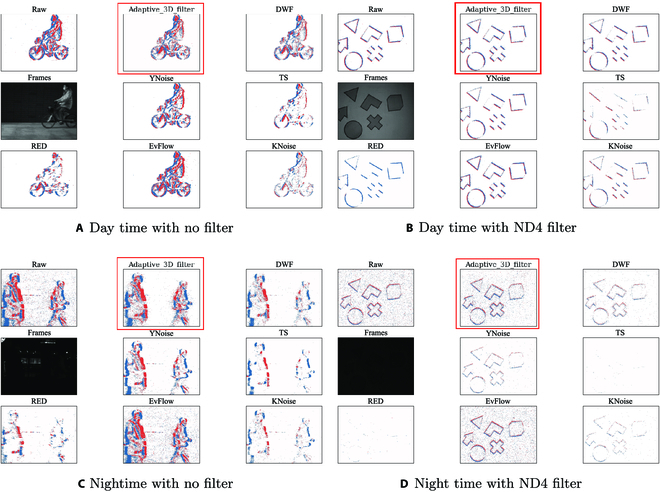
Comparative visual analysis of denoising algorithms. This figure illustrates a range of scenarios: (A) a bicycle in daytime lighting; (B) multiple fast-moving objects in a clear, noise-free daytime setting, testing the speed sensitivity of the denoisers; (C) nonrigid body motion captured under nighttime street lights; and (D) various fast-moving objects in a dark, nighttime environment. We marked ours in red.

Upon a detailed qualitative analysis of Fig. 5, we discern the performance characteristics of various denoising algorithms across different lighting conditions.

In Fig. 5A, under daytime lighting with no filter, it is observed that while all algorithms manage to reduce noise to a degree, there is a notable variance in their propensity to overdenoise. Algorithms such as TS and KNoise appear to excessively smooth the event data, resulting in a loss of critical structural detail within the bicycle image. Conversely, our adaptive 3D filter and EvFlow demonstrate a more conservative denoising approach, retaining more of the scene’s inherent details and avoiding the pitfalls of overdenoising.

In Fig. 5B introduces an ND4 filter, simulating reduced light conditions. In this setting, the challenge of balancing noise suppression with detail preservation becomes more pronounced. Algorithms like RED and TS begin to exhibit clear signs of overdenoising, as evidenced by a substantial diminution of event density, suggesting an aggressive loss of information. Our adaptive 3D filter, however, maintains a superior level of detail retention, indicating its robust performance under suboptimal lighting conditions.

As we transition to nocturnal conditions in Fig. 5C, without a filter, the inherent noise levels in the data increase significantly. This increment in noise presents a heightened challenge for denoising algorithms. Here, we observe that algorithms like RED and TS tend to overdenoise, leading to an important loss of features. This is particularly problematic for capturing the nuances of nonrigid body motion under street lights. Our adaptive 3D filter continues to mitigate noise effectively while preserving the critical features necessary for accurate event interpretation.

Under the most challenging condition, Fig. 5D, under nighttime with an ND4 filter, the algorithms are subjected to increased noise levels. Many algorithms exhibit decreased performance, with some, such as TS and RED, completely overdenoising the scene, resulting in the obliteration of essential event information. Moreover, DWF and YNoise display pronounced tendencies toward excessive denoising. In contrast, our adaptive 3D filter adeptly preserves the structural integrity of fast-moving objects, demonstrating its capacity to maintain high-quality event data without succumbing to overdenoising.

Throughout these analyses, it is evident that our adaptive 3D filter and EvFlow maintain a consistently higher standard of denoising across both daytime and nighttime conditions. They effectively reduce noise while safeguarding the detail integrity of the event data, showcasing their advanced capabilities in event denoising without overdenoising, which is critical for retaining essential information within dynamic environments. However, the quantitative analysis reveals that EvFlow’s CT exceeds that of our algorithm by a factor of 145, underscoring the superior efficiency of our approach.

## Discussion

In this study, we explored the impact of varying ambient lighting on noise generation in event cameras, marking a pioneering analysis in this field. Our investigation uniquely focuses on the interaction between natural and artificial light sources and the camera’s sensor performance. We observed that intense natural light, such as bright sunlight, often leads to pixel saturation in the camera’s sensor. This saturation diminishes the sensitivity of the pixels, adversely affecting the quality and reliability of the event data captured. Conversely, under artificial lighting in low-light outdoor settings, we noted significant spatial disparities in the event data. These disparities present challenges for clustering algorithms like HDBSCAN, particularly in terms of accurately grouping data points. To further understand this dynamic, we also varied the camera’s frame velocity across different lighting conditions. This approach enabled us to establish a correlation between the camera’s velocity and the pixel activity, which we refer to as PA. Our findings show that the PA metric effectively captures the dynamism of the surroundings, regardless of lighting conditions.

After calculating the noise frequency, our investigation revealed that the noise predominantly resides in the low-frequency range (see the “Noise power density spectral analysis” section). This discovery was pivotal in shaping the development of our bioinspired adaptive denoising algorithm. Drawing from the mechanisms of the human visual system, particularly the way motion is processed in the retina as detailed in the “Comparison to the human visual system” section, we tailored our algorithm to this specific characteristic of the noise. In fact, in the human retina, certain cells are dedicated to motion perception, adjusting their filtering based on environmental activity levels. These cells are adept at reducing static, low-frequency background signals in highly dynamic scenes, thereby focusing on the more critical, high-frequency signals such as moving obstacles or significant scene changes. These high-frequency elements are crucial for the brain’s rapid processing and response mechanisms. Applying this biological principle, our adaptive denoising algorithm is designed to function primarily as a high-pass filter. It specifically targets and attenuates the low-frequency noise components that we identified in our analysis, while crucially preserving the high-frequency details of the scene. The algorithm dynamically modulates its filtering parameters in response to the PA, replicating the retina’s capability to adjust to different levels of dynamism in the environment. By adopting this approach, our algorithm effectively processes the data from event cameras, ensuring that vital high-frequency information is retained and emphasized. This strategy not only improves the fidelity of the captured scene data but also aligns closely with the sophisticated natural processes of the human visual system, particularly in how it manages and interprets motion.

In our research, we recognize a fundamental challenge in our adaptive denoising algorithm: distinguishing between authentic low-frequency events (the ground truth) and low-frequency noise. Despite the algorithm’s overall success in preserving critical features in the dataset, it sometimes incorrectly classifies genuine, slowly evolving scene changes as noise. This limitation leads us to more accurately describe our denoising algorithm as a data compression algorithm, acknowledging the potential for information loss, particularly in the lower-frequency range. However, this information loss varies with the camera’s velocity. At higher velocities, even stationary backgrounds produce high-frequency components due to the relative motion between the camera and the scene. This results in the enhanced detection of both the static background’s edges and moving obstacles, as depicted in Fig. 4. The principle here is that increased camera velocity heightens the dynamic nature of the environment, thereby enhancing the algorithm’s ability to differentiate between low-frequency noise and high-frequency components, including static backgrounds and moving objects. Conversely, when the camera moves slowly or is hovering, the algorithm faces a different challenge. Although our adaptive filter accounts for the less dynamic environment by modifying the clustering parameters in our unsupervised learning algorithm, it still tends to prioritize higher frequencies. To mitigate the potential loss of low-frequency information in these scenarios, we demonstrate that continuous event generation by any object over time ensures its persistence within the cluster, as defined by the adaptive parameters of our 3D clustering algorithm, as illustrated in Fig. 5. This approach reflects a balance between data compression and the preservation of significant scene elements. The algorithm’s capability to adapt its filtering and clustering strategies based on the camera’s movement and the surrounding environment’s dynamism exemplifies an advanced level of sophistication in event data processing. By doing so, it maintains a focus on capturing and retaining crucial scene changes while effectively managing the inherent trade-offs in noise reduction and data compression.

To further elucidate our approach, let us consider a specific example within the operational context of our adaptive denoising algorithm. Suppose that there is an object within the field of view of an event camera that is moving at a similar velocity to the camera itself, which is also moving at a low velocity. When the object and the camera are moving in parallel directions, the relative velocity between them is negligible. In this case, the object does not represent a collision threat due to the alignment of their navigation paths. Our algorithm, in this scenario, demonstrates a deliberate trade-off by not flagging the object as a significant element. This decision aligns with our goal to selectively focus on more dynamically relevant changes and potential threats in the environment, which is not only a cost-effective strategy but also akin to the human brain’s approach in prioritizing visually significant elements over static or nonthreatening aspects. Conversely, if the object moves in a direction that is different from the camera’s but at a comparable velocity, the relative velocity within the camera’s frame of reference changes. This change is detected by our algorithm, which is designed to be sensitive to variations in relative motion, thereby recognizing the object as a potential point of interest or threat. This capability underscores the algorithm’s effectiveness in responding to changes in the scene, especially those with implications for navigation and safety.

In summary, our adaptive denoising algorithm’s ability to discern relative motion and directionality within its environment is crucial. It allows for efficient and effective processing of event data, focusing on genuinely significant changes while minimizing unnecessary detections. This approach exemplifies a sophisticated balance between data compression, noise reduction, and the preservation of critical environmental information.

## Materials and Methods

### Noise power density spectral analysis

To enhance the precision and consistency of event camera systems, it is essential to comprehend the types and occurrences of noise that can affect the recorded data. In this section, we will undertake an in-depth analysis of the predominant sources of noise in event cameras and compute their respective power spectral densities. This quantification of noise power density will offer insights into the frequency bands most significantly influenced by noise interference. Moreover, we will conduct an evaluation of how various illumination conditions contribute to the overall noise level.

#### Temporal noise

Temporal noise, also referred to as shot noise, is a form of noise that originates from the unpredictable arrival times of charge carriers, such as photons or electrons [24]. Shot noise in event cameras is attributed to the intrinsic randomness of photon arrival times at the photodetector, which results in the fluctuation of signal amplitude. Thus, when light is incident on a pixel’s photoreceptor as seen in Fig. 1B, photons are absorbed, generating electron-hole pairs that contribute to the photocurrent. Since the arrival times of photons follow a Poisson distribution [25], the resulting photocurrent *I*_ph_(*t*) exhibits fluctuations as seen in Fig. S1B, which are referred to as shot noise:

$$I_{\text{ph}}\left(t\right)={\bar{I}}_{\text{ph}}+i_{\text{shot}}\left(t\right)$$ (2)

where ${\bar{I}}_{\text{ph}}$ is the average photocurrent and *i*_shot_(*t*) is the time varying current noise. The expression of the noise is given by [26]:

$$|i_{\text{shot}}^{2}\left(t\right)|=\bar{i_{\text{shot}}^{2}}=2q{\bar{I}}_{\text{ph}}\Delta f$$ (3)

where *q* = 1.602176634 × 10^−19^ C is the electrical charge carried by a proton and Δ*f* is the frequency range.

The shot noise of photocurrent, *i*_shot_(*t*), can be quantified by calculating its noise power, which is expressed in terms of the current noise spectral density, *S_i_*. The current noise spectral density due to shot noise is given by:

$$S_{i}=\frac{\bar{i_{\text{shot}}^{2}}}{\Delta f}=2q{\bar{I}}_{\text{ph}}$$ (4)

When a current signal passes through an impedance *Z*(*f*), the voltage signal generated by the current noise across the impedance is given by Ohm’s law *v*_shot_(*f*) = *i*_shot_(*f*)*Z*(*f*). Thus, the PSD of the voltage noise *S*_v_:

$$\begin{array}{c} S_{v}\left(f\right)=\frac{\bar{v_{\text{shot}}^{2}}}{\Delta f}\\ \;=\frac{\bar{i_{\text{shot}}^{2}}}{\Delta f}{\left|Z\left(f\right)\right|}^{2}\\ S_{v}\left(f\right)=S_{i}{\left|Z\left(f\right)\right|}^{2} \end{array}$$ (5)

The pixel circuitry as represented in Fig. 1 is composed of 2 capacitors *C*_1_ and *C*_2_ in series. Thus, the impedance equation *Z*(*f*) is:

$$Z\left(f\right)=\frac{1}{j2\pi fC_{\text{total}}}$$ (6)

where *C*_total_ = 1/*C*_1_ + 1/*C*_2_ and *f* is the frequency.

From Eqs. 4 to 6, the PSD of the voltage temporal noise *S*_v_, as a function of frequency *f* is:

$$S_{v}\left(f\right)={\left(\frac{1}{2\pi fC_{\text{total}}}\right)}^{2}\left(2q{\bar{I}}_{\text{ph}}\right)$$ (7)

#### Thermal noise

In a circuit with 2 capacitors in series without any resistance, the thermal noise arises from the parasitic resistance within the capacitors themselves, referred to as the equivalent series resistance (ESR) [27]. Each capacitor (*C*_1_ and *C*_2_) has an associated ESR (*R*_1_ and *R*_2_), which can be modeled as a resistor in series with the capacitor as seen in Fig. S1A. The circuit can then be considered as 2 resistor–capacitor (RC) branches connected in series. The total ESR *R*_total_ = *R*_1_ + *R*_2_ is the sum of the individual ESRs.

The thermal noise voltage *v*_thermal_ in this context can be calculated using the Johnson–Nyquist noise formula [28]:

$$v_{\text{thermal}}\left(f\right)=\sqrt{4kR_{\text{total}}\Delta f}$$ (8)

where *k* = 1.38 ∗ 10^−23^ J/K is the Boltzmann constant, *T* is the temperature in kelvin, *R*_total_ is the total resistance in ohms, and Δ*f* is the bandwidth.

The thermal noise current *i*_thermal_(*f*) is calculated by dividing the noise voltage *v*_thermal_(*f*) by the total impedance *Z*_total_(*f*) of the equivalent circuit:

$$i_{\text{thermal}}\left(f\right)=\frac{v_{\text{thermal}}\left(f\right)}{Z_{\text{total}}\left(f\right)}$$ (9)

where *Z*_total_ = (−*j*/(2*πfC*_1_)) + *R*_1_ + (−*j*/(2*πfC*_2_)) + *R*_2_ is the equivalent impedance of the 2 capacitors and 2 resistors in series.

Since the capacitors are in series, the noise current will be the same for both capacitors. The noise voltage across each capacitor (*v*_*nC*_1__ and *v*_*nC*_2__ ) can be calculated using Ohm’s law:

$$\begin{array}{ll} v_{nC_{1}}\left(f\right) & =i_{\text{thermal}}\left(f\right)Z_{C_{1}}\left(f\right)\\ v_{nC_{2}}\left(f\right) & =i_{\text{thermal}}\left(f\right)Z_{C_{2}}\left(f\right) \end{array}$$ (10)

where *Z*_*C*_1__(*f*) =  − *j*/(2*πfC*_1_) and *Z*_*C*_2__(*f*) =  − *j*/(2*πfC*_2_) are the individual capacitor impedances. To find the total noise voltage power density, the power across each capacitor (*S*_*v_n_C*_1__ and *S*_*v_n_C*_2__) needs to be calculated:

$$\begin{array}{ll} S_{v_{n}C_{1}}\left(f\right) & =\frac{{\left|v_{nC_{1}}\right|}^{2}}{\Delta f}\\ S_{v_{n}C_{2}}\left(f\right) & =\frac{{\left|v_{nC_{2}}\right|}^{2}}{\Delta f} \end{array}$$ (11)

Thus, to find the total thermal voltage noise power density *S*_*v_n_*Total_ as function of *f*, the power of both capacitors is summed and divides it by Δ*f*:

$$\begin{array}{c} \begin{array}{ll} S_{v_{n}\text{Total}}\left(f\right) & =\frac{S_{v_{n}C_{2}}+S_{v_{n}C_{2}}}{\Delta f}\\ S_{v_{n}\text{Total}}\left(f\right) & =\left(\left(\frac{4kR_{\text{total}}}{{\left(R_{\text{total}}2\pi fC_{1}\right)}^{2}+\alpha_{1}}\right)+\left.\frac{4kR_{\text{total}}}{{\left(R_{\text{total}}2\pi fC_{2}\right)}^{2}+\alpha_{2}}\right)\right) \end{array} \end{array}$$ (12)

where *α*_1_ = (1 + *C*_1_/*C*_2_)^2^ and *α*_2_ = (1 + *C*_2_/*C*_1_)^2^.

#### Intrinsic noise

Intrinsic noise in event cameras originates from internal hardware factors, with 2 notable sources being leak noise and hot-pixel noise. Leak noise stems from spontaneous ON events, while hot-pixel noise is a result of persistently overactive pixels within the sensor. In this context, DVS pixels can encounter spontaneous ON events, or leak events, exhibiting a polarity of +1. These events transpire at a rate of 0.1 Hz [10] and are predominantly induced by reverse diode leakage currents in reverse-biased p–n junctions between the deep n-well and source–drain node [19], in addition to parasitic photocurrents from the change detector reset switch. It is important to note that leak events do not display temporal or spatial correlation with genuine generated events, underscoring their unique nature and the necessity for meticulous examination in DVS system analysis and optimization.

In addition, DVS systems can present a small proportion of pixels (approximately 0.7% [19]) with irregular firing rates due to hardware interference, referred to as hot pixels, as depicted in Fig. S2A. Hot pixels can induce high-frequency burst events and elevated noise in the output of DVS systems. To minimize the incidence of false event detection and the associated high-frequency noise, researchers have recommended implementing a refractory period, during which a pixel remains inactive for a specified duration after its previous response, thereby reducing false event detection and the corresponding high-frequency noise [19].

#### Noise power density spectral distribution and the impact of lighting on noise

The analysis of PSD plots, as seen in Fig. S2B, for dominant voltage noises provides a comprehensive understanding of noise behavior, which, in turn, enables the optimization of filter design to address noise within the appropriate frequency range. In the case of temporal noise, the voltage noise spectral density *S*_v_(*f*) (Eq. 7) increases as the frequency decreases. This behavior is frequently observed in circuits containing capacitive elements, where low-frequency noise is often more pronounced because of the increased impedance of capacitors at lower frequencies. Regarding thermal noise, the frequency in the voltage noise spectral density *S*_*v_n_*Total_(*f*) is multiplied by the small *R*_total_, rendering the denominator in Eq. 12 constant and equal to *α*_1, 2_. As a result, for low frequencies, the power density of thermal noise remains constant and only begins to decrease beyond a certain threshold when the frequency surpasses the diminutive value of *R*_total_. This observation emphasizes the importance of understanding the technical aspects of noise behavior to effectively design and optimize filters for noise attenuation within the appropriate frequency spectrum.

In neuromorphic cameras, the impact of certain noise types is also correlated with the extent of low-light conditions. Specifically, in dim light situations, the number of incident photons decreases compared to normal lighting conditions (less than 10^−2^ photons·μ^−2^·s^−1^ for starlight condition compare to greater than 10^8^ photons·μ^−2^·s^−1^ for bright sunlight [29]). As a result, the photocurrent *I*_ph_ produced in the photodetector is reduced, regarding the lower quantity of incident photons. This decreasing in signal level renders the system more sensitive to fluctuations. Thus, under these low-light conditions, shot noise (temporal noise), arising from the random arrival of photons, gains prominence. In fact, with fewer photons present in dim light situations, the accurate detection of brightness changes becomes more challenging as the signal-to-noise ratio of the system decreases significantly. Contrary to temporal noise, intrinsic and thermal noise are not directly affected by the brightness of the light. Instead, their origins lie in the camera’s internal electronic components and their physical properties. In addition, in bright light, *I*_ph_ generated in the photodetector is larger, resulting in a higher signal level. With an increased signal strength, the system can better tolerate the presence of all type of noise, leading to a higher signal-to-noise ratio.

### 3D adaptive unsupervised learning-based filter

#### 3D time-discrete signal representation for noise filtering

Event cameras represent a profound departure from conventional imaging paradigms, emphasizing changes in the visual scene over static intensity values. These devices operate in a 3D space *ℝ*^3^ defined by spatial coordinates (*x*, *y*) and temporal coordinate *t*, presenting unique challenges and opportunities. In this context, when one considers the idea of converting this event stream into time images, the endeavor to make the data compatible with traditional image processing algorithms might inadvertently compromise its temporal integrity. The richness and granularity that event-based data offers could be blurred, obscuring rapid shifts and subtleties in the visual field. Any event that unfolds between 2 slices might be averaged out, thereby potentially misrepresenting or even omitting swift luminance changes, diminishing the fidelity that the native stream would otherwise convey. Further exacerbating this issue, the noise-filtering process becomes particularly challenged. In the native stream, transient noise events are discernible because of their isolated temporal occurrence. However, in time images, these sporadic noise events might get integrated into the visual representation, possibly interpreted as genuine scene changes. Consequently, noise-filtering algorithms applied to these images may either fail to detect such artifacts or, in their attempts to rectify them, might unintentionally diminish genuine rapid scene transitions that manifest temporally rather than spatially. Such misrepresentations not only hamper the true depiction of a dynamic environment but can also lead to cascading inaccuracies in downstream processing tasks, undermining the inherent advantage of event-based vision systems.

#### Comparison to the human visual system

In dissecting the challenges of event-based data processing, the compelling analogies offered by the retina’s biological intricacies can be drawn. RGCs work with a discernment intricately rooted in their biophysical properties. Their response patterns are shaped by meticulously orchestrated ion channel dynamics, coupled with modulator inhibitory inputs from interneurons. These mechanisms establish precise firing thresholds, such that only stimuli with intensities within a certain period of time surpassing these thresholds elicit action potentials. This selective mechanism ensures that RGCs can underscore salient intensity changes in the visual scene while effectively sidelining inconsequential noise. Drawing a parallel to the computational realm, the 3D HDBSCAN algorithm’s core tenets echo these selective principles of the retina. At the heart of 3D HDBSCAN is the core distance, a metric analogously setting a benchmark for cluster determination. Just as only potent stimuli lead to RGC responses, only data regions with densities along the time axis, surpassing this core distance are designated as bona fide clusters. Moreover, HDBSCAN’s nimbleness, manifested in its ability to detect clusters of disparate densities in heterogeneous data, mirrors the adaptive thresholds seen in neural entities. In response to fluctuating input patterns, both neural circuits and 3D HDBSCAN can dynamically adapt to the situation. Indeed, neurons might modulate their sensitivity through processes like synaptic plasticity, while 3D HDBSCAN can adjust parameters in tandem with the prevalent event density in the data landscape by retrain to fit the new data.

Delving deeper into the retinal architecture unravels a sophisticated interplay of cellular components and processes. The retina does not merely operate through isolated neural entities but rather a meticulously coordinated cascade of signal processing from a population of neurons. Crucially, these neurons exhibit adaptive behavior in their responses. Their activity is not static but dynamically modulates based on local (neighbor–neuron interactions) and global (overall network dynamics) stimuli. For instance, under high-luminance conditions (high neuronal activity), mechanisms like synaptic depression and altered ion channel conductances kick in, modifying the neuron’s sensitivity and responsiveness to prevent saturation. This ensures that the retina remains a reliable sensory organ across a vast range of environmental light conditions. Drawing parallels with this adaptive retinal machinery, our method’s functionality is not rigid. It does not only cluster data points based on a fixed set of parameters but also evaluates data contextually. In scenarios where the overall event density of the data is high, 3D HDBSCAN can dynamically adjust its input parameters, particularly the core distance and minimum samples, regarding its PA. This adaptability ensures that the algorithm does not group too many events into an oversized cluster or fail to discern between proximate but distinct clusters. By aligning its clustering thresholds with the prevalent data density, much like how retinal neurons adapt their thresholds to ambient luminance, HDBSCAN showcases an inherent a context-awareness, ensuring optimal performance across a diverse array of data landscapes.

Finally, in the comprehensive findings elucidated in our results section, our approach showcases a notable proficiency in discerning and subsequently negating persistent low-frequency events, which predominantly correspond to the ambient, static background. This step is instrumental in our advanced data compression protocol. The underpinnings of this process are deeply rooted in the intricate neurophysiological processes of the retina. The mammalian retina uses a combination of temporal filtering and spatial antagonistic receptive fields. The horizontal cells, a different type of retinal interneuron, use a phenomenon known as lateral inhibition. By inhibiting adjacent photoreceptors in response to intense light stimulation, they fine-tune and enhance the contrast at visual edges, serving as a sort of spatial high-pass filter. This mechanism ensures that static, unchanging stimuli produce diminished responses over time, while transient or changing stimuli are accentuated. RGCs, which are the output neurons of the retina, further integrate these signals and relay them to the brain. Through a cascade of excitatory and inhibitory synaptic interactions, the retina effectively emphasizes rapid changes in luminance or contrast while suppressing consistent, unchanging inputs. Building on these insights, our computational method integrates temporal differentiation mechanisms, akin to the temporal filtering in retinal cells, to efficiently isolate events that represent dynamic changes in the visual scene. In other words, our approach effectively suppresses the low-frequency, static components of the data. This biologically inspired methodology ensures that while the consistent, redundant background is efficiently compressed, the salient and dynamic event details are retained with high fidelity. In essence, by integrating principles from retinal neuroscience, we have crafted a compression technique that emphasizes the dynamic and reduces the redundant, optimizing the representation for subsequent analytical tasks.

#### Algorithm design and framework

Our strategy borrows foundational principles from biological systems, specifically the human visual system as seen in the section above. Indeed, signal processing incorporates both spatial and temporal summation [30]. Spatial summation is a process wherein signals from multiple cells are synthesized into a singular output, while temporal summation refers to the integration of these signals over a defined temporal scale. These operations enhance photoreceptivity and amplify the signal-to-noise ratio. Considering the analogy between the function of pixels in event cameras and the role of RGCs, we model the event stream as a 3D discrete signal, with due emphasis on the temporal dimension. This model offers an avenue for the use of advanced signal processing techniques, thereby augmenting the efficacy of the denoising process.

Incorporating the 3D HDBSCAN [31], an unsupervised machine learning algorithm, offers a novel bioinspired approach for event data processing. This method interprets events as 3D noisy signals, allowing for effective clustering along the temporal axis. This technique leverages the high temporal resolution and dynamic nature of event streams, enabling efficient processing of larger event quantities within an extended time window. HDBSCAN operates with 2 primary parameters: MinPts, defining the minimum number of points required to form a dense region, and MinClusterSize, setting the smallest size grouping considered meaningful. Unlike DBSCAN [32], HDBSCAN constructs a hierarchy of clusters without a need for a global distance parameter (*ϵ*). It is used instead of DBSCAN particularly when datasets present significant density variations introduced by factors such as artificial lights. By adjusting these 2 parameters, the sensitivity of the clustering process can be finely tuned, enabling more effective identification of event clusters. Indeed, as seen in Algorithm 1, first, the events structure is transformed to “spikes” to calculate PA. The spike matrix is a representation of the event data transformed into a binary form known as spikes, effectively mapping the spatiotemporal event data onto a grid. The spike matrix will have dimensions of the total number of pixels (height by width) by number of time steps. The value at a specific position [*i*, *j*] in the matrix indicates whether the pixel *i* has fired (has a spike) at the time step *j*. Although it is represented in a 2D matrix, the spike matrix is conceptually a 3D structure. The (*x*, *y*) coordinates of each pixel are converted to a column index, and the time steps represent the temporal dimension. Thus, each entry in the spike matrix can be seen as the state (fired or not fired) of a pixel at a specific point in time. Upon computation of the “spikes”, the evaluation of PA can be performed. In the context of neuroscience, PA refers to the coordinated firing or activity patterns exhibited by a collective group of neurons within a specific region or network in the brain. Importantly, PA provides a significant understanding of the motion perceived by the retina. The collective neuronal firing patterns can encode information about the direction, speed, and complexity of moving objects within the visual field [33]. For example, a surge in PA might correspond to rapid movements in the environment, while a steady, low-level activity could be indicative of stationary or slow-moving objects. The precise patterning and timing of neuronal firing provide a dynamic and nuanced representation of the moving elements within the environment. This offers a deeper understanding of how motion is encoded and interpreted in the visual processing system. In this context, the calculation of PA, expressed in hertz, can be carried out as follows:

$$\text{PA}\left(t\right)=\frac{n\left(t,\;\;t\;\;+\;\;\Delta t\right)}{N\Delta t}$$ (13)

where *t* is the time stamp, *n* is the number of pixels firing at each time step Δ*t*, and *N* is the total number of pixel.

Upon computing the PA, adjustments to the parameters MinPts and MinClusterSize are made to better align with the environmental dynamics captured by the event camera and the observed data sparsity. When PA values are low, it suggests a sparsity in the event data, commonly seen in environments with little to no motion. Conversely, as PA values rise, it indicates the event camera’s operation in a lively, motion-intensive environment. In such contexts, the parameters MinPts and MinClusterSize are dictated by the function *f*(PA), described as *f* = *λ* ∗ PA. Here, *λ* serves as an adjustable constant, fine-tuning the denoising process. The function *f*(PA) is designed to scale the unsupervised machine learning algorithm parameters in relation to the PA. In dynamic settings filled with motion, event cameras relay denser data, logging events that are spatially and temporally proximate. Thus, elevating the values of these parameters in such scenarios ensures precise clustering, reducing the risk of unrelated events being mistakenly clustered due to data density. However, when the function *f*(PA) decreases, it signifies a need to accommodate for less dense data, requiring a reduction in the HDBSCAN’s parameters to ensure that genuine clusters are not dismissed or overlooked.

This adaptability ingrained in the HDBSCAN algorithm allows it to respond aptly to changes in environmental dynamics and data sparsity, resulting in a context-sensitive and more accurate denoising process


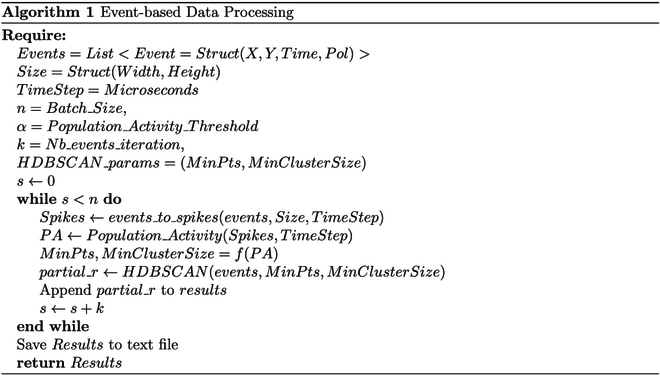


## Experimental design

In the data acquisition phase of our experimental framework, the dynamic and active pixel vision sensor (DAVIS346) from iniVation [34] was used to record the same location under 3 distinct lighting conditions: dark (nighttime), dim (late evening), and bright (noon). The DAVIS346 sensor was selected for its outstanding temporal resolution, extensive dynamic range, and its capacity to adeptly handle high-contrast and rapid-motion scenarios.

For each of these lighting conditions, recordings of the same oudoor spot were conducted at 2 distinct speeds: a low speed of 4.5 km/h and a high speed of 8.5 km/h. We also introduced rotational movements, which enhances our understanding of sensor responsiveness to motion changes. This approach was designed to mimic the variations in motion dynamics that the sensor might encounter in real-world applications. By maintaining consistent speeds across different lighting conditions, we aimed to isolate and study the sensor’s response to changing illumination levels.

This meticulous setup resulted in a total of 6 unique datasets, each with a duration of 1 min and 30 s. Specifically, for each of the 3 lighting conditions, there were 2 datasets corresponding to the 2 different speeds (low and high), resulting in pairs like “dark slow velocity” and “dark fast velocity”, “dim light slow velocity” and “dim light fast velocity”, and “bright slow velocity” and “bright fast velocity”. Following the data collection phase, these datasets were processed offline.

## 1D discrete FFT

In the transformation process of the event data to a structured format, the spatiotemporal event data are mapped onto a grid to form a spike matrix. Each row of this matrix represents a single pixel of the sensor, while the columns correspond to discrete time steps. Mathematically, the spike matrix *S* is defined as follows: *S*(*i*, *j*) = 1 if pixel *i* has generated an event (or “fired”) at time step *j*, and *S*(*i*, *j*) = 0 otherwise. In essence, *S* is a binary matrix of dimensions *N* × *T*, where *N* is the total number of pixels and *T* is the number of time steps. Each element *S_ij_* in this matrix can be interpreted as the state (fired or not fired) of a pixel *i* at a specific point in time *j*:

$$S\left(i,j\right)=\left\{\begin{array}{cc} 1 & \text{if pixel}\;i\;\mathrm{has}\;\text{generated an event}\;\mathrm{at}\;\text{time step}\;j,\\ 0 & \text{otherwise} \end{array}\right.$$ (14)

To analyze the temporal dynamics of each pixel, a 1D FFT is applied to each row of the spike matrix, converting the time-domain signal of each pixel into the frequency domain. The FFT for each pixel *i* can be mathematically represented as:

$$F\left(i,\;\;k\right)=\sum_{j=0}^{T-1}‍S\left(i,\;\;j\right)\cdot e^{-\frac{2\text{πijk}}{T}}$$ (15)

where *F*(*i*, *k*) is the *k*th frequency component of the FFT for pixel *i* and *k* ranges from 0 to *T* − 1.

Following the FFT, the PSD for each pixel is computed, providing a measure of the power distribution over different frequency components for each pixel’s activity. The PSD for pixel *i* at frequency *k* is given by:

$$\text{PSD}\left(i,k\right)=\frac{{\left|F\left(i,k\right)\right|}^{2}}{T}$$ (16)

This analysis, executed independently for each pixel in the sensor, provides a detailed characterization of the temporal patterns and periodicities inherent in the event data. Indeed, in signal processing, the FFT is a widely used technique for noise removal [35]. In the context of event camera data, applying the FFT to each pixel’s temporal activity, represented as a sequence of spike events, reveals the frequency components of that activity. The PSD, computed as the square magnitude of the FFT, helps identify significant frequencies and distinguish signal from noise [36].

## Data Availability

For access to the recording of the 6-event dataset captured using the event camera DAVIS346 as detailed in the “Experimental design” section, please email the corresponding author (Y.L.). They will provide the necessary information and links to obtain the data.
