# Supplementary material for: Adaptive Unsupervised Learning-Based 3D Spatiotemporal Filter for Event-Driven Cameras
Source: Research (Wash D C). 2024 Apr 1;7:0330. doi: 10.34133/research.0330 (PMC10981976; doi:10.34133/research.0330)
Supplement: Supplementary 1 — Figs. S1 to S4 Movies S1 and S2 [file research.0330.f1.zip › Supplementary Materials.docx]

**Supplementary Materials**

**Figures**

Fig. S1 Event Camera Pixel Circuitry and Photocurrent Noise Analysis:

(a) Equivalent event camera’s pixel circuit with the associated ESR (b) The effect of shot noise on time-varying photocurrent Iph(t), showing the average photocurrent Iph and the associated temporal noise level in(t) .


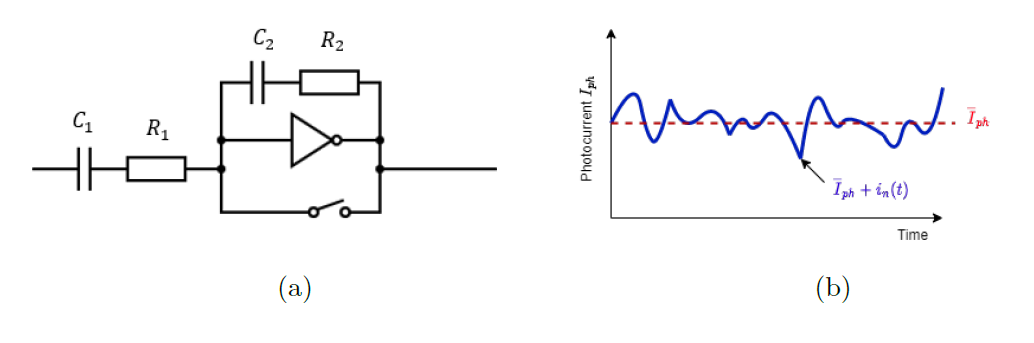


Fig. S2 Hot Pixel Behavior and Voltage Noise Power Density Analysis:

(a) A hot pixel in a camera refers to a specific pixel that consistently fires and produces events during a short period of time without real change in brightness. In the graph, axis x and y refer to the (x,y) coordinates of each pixel in the DVS camera (b) Voltage Noise power density distribution across frequencies. The blue line refers to the thermal voltage (Eq. 12) and the red line to the temporal noise (Eq.7).


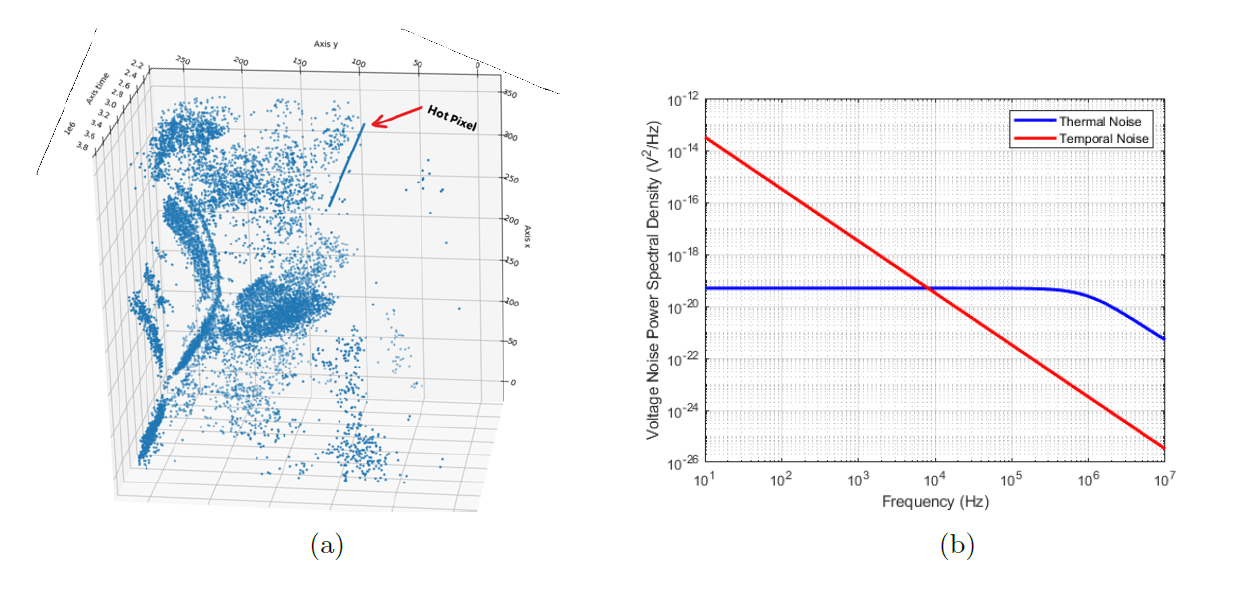


Fig. S3 Mean Population 949 Activity for different natural light conditions and different velocities Computed and Plotted in 10-Microsecond Intervals per Iteration:

(a) Nighttime , Slow Velocity (b) Nighttime, High Velocity (c) Dim Light, Slow Velocity (d) Dim Light, High Velocity (e) Bright Light, Slow Velocity (f) Bright Light, High Velocity


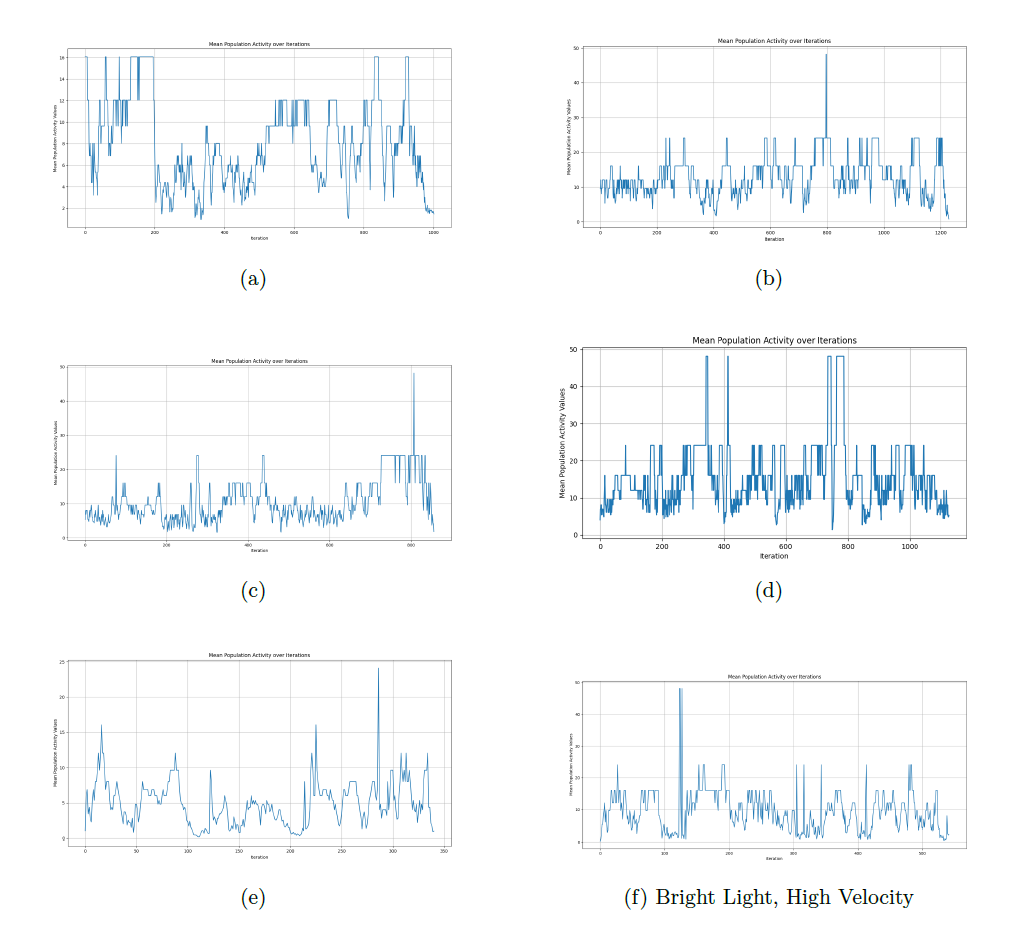


Fig. S4 De-noising Algorithm Performance for slow moving camera: Clustering. (a)Clusters Unfiltered events - Bright light (b) Cluster Filtered events - Bright light (c) Unfiltered Events (d) Filtered events


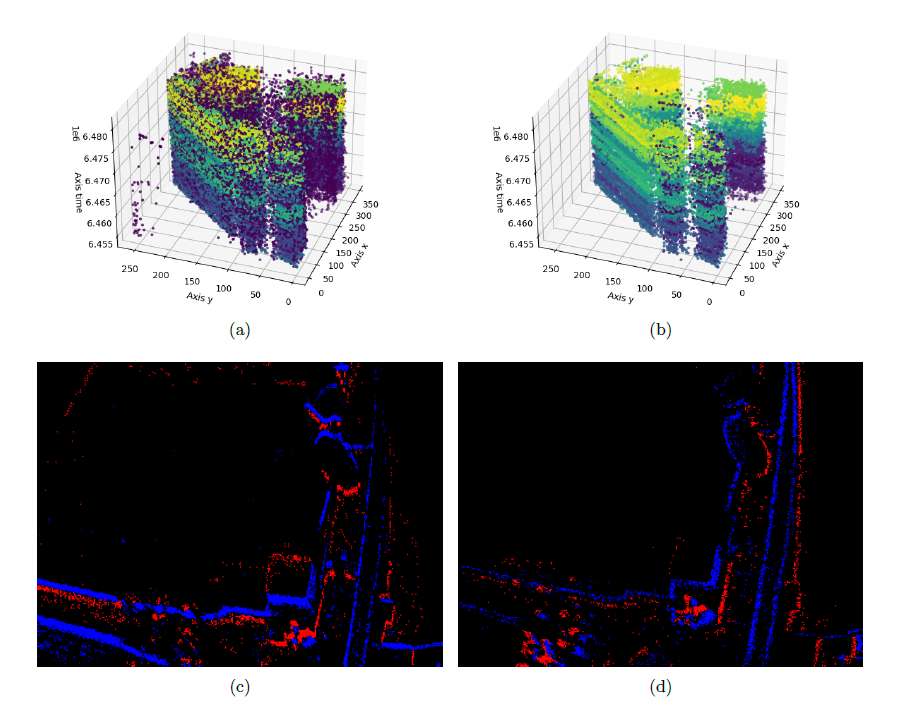


**Other Files**

3d adaptive filtering.mp4

Benchmark denoising.mp4
